# S149. EFFECTS OF INTRANASAL OXYTOCIN ON RESTING CEREBRAL BLOOD FLOW IN PEOPLE AT ULTRA-HIGH RISK FOR PSYCHOSIS

**DOI:** 10.1093/schbul/sby018.936

**Published:** 2018-04-01

**Authors:** Cathy Davies, Grazia Rutigliano, Marco Cappucciati, Andrea De Micheli, Valentina Ramella-Cravaro, Umberto Provenzani, Andre Schmidt, Yannis Paloyelis, Philip McGuire, Paolo Fusar-poli

**Affiliations:** Institute of Psychiatry, King’s College London

## Abstract

**Background:**

Recent research suggests that individuals at ultra-high risk for psychosis (UHR) show altered resting cerebral blood flow (rCBF) in key regions linked to psychosis pathophysiology: the hippocampus, midbrain, and basal ganglia. Greater perturbations in basal ganglia rCBF were correlated with positive psychotic symptoms, while remission from the UHR state was associated with a longitudinal normalization of hippocampal rCBF. Oxytocin -a neuropeptide with potential anxiolytic and prosocial properties- is currently under investigation as a novel therapeutic for a number of neuropsychiatric disorders. Previous work conducted in healthy males demonstrated that a single acute dose of intranasal oxytocin had marked effects on rCBF across all of the aforementioned regions (hippocampus, basal ganglia, midbrain), as well as the amygdala, anterior cingulate cortex and cerebellum - regions where neurofunctional alterations have been previously reported in UHR groups. Despite these findings, no studies have yet examined the effects of intranasal oxytocin on resting perfusion in UHR individuals.

**Methods:**

In a double-blind, placebo-controlled, crossover design, 30 UHR males underwent two MRI scans at 3 Tesla, once after 40IU intranasal oxytocin and once after matched placebo (one-week wash-out). Arterial spin labeling (ASL) was used to measure rCBF starting approximately 22 minutes post-intranasal administration. The severity of attenuated psychotic symptoms was assessed using the Comprehensive Assessment of At-Risk Mental States (CAARMS). Measures of social cognition, emotional processing and level of functioning were also acquired. We hypothesized that relative to placebo, a single acute dose of intranasal oxytocin would modulate rCBF in the hippocampus, basal ganglia and midbrain, and that this effect would be greater in those with more severe baseline deficits in social and emotional functioning.

**Results:**

Data analysis is currently ongoing and the results will be presented at the conference.

**Discussion:**

These results will provide physiological evidence for a potential first-in-class intervention for UHR patients. Given the current lack of evidence for effective treatments in this patient group, better understanding of the neural correlates of the high-risk state and the physiological basis for the effects of novel therapeutics is desperately warranted.

